# Supplementary material for: Hypoxia induced responses are reflected in the stromal proteome of breast cancer
Source: Nat Commun. 2023 Jun 22;14:3724. doi: 10.1038/s41467-023-39287-7 (PMC10287711; doi:10.1038/s41467-023-39287-7)
Supplement: Supplementary file 1 — Supplementary Information [file 41467_2023_39287_MOESM1_ESM.pdf]

SUPPLEMENTARY INFORMATION

Hypoxia induced responses are reflected in the stromal proteome in breast cancer

Silje Kjølle[1], Kenneth Finne[1], Even Birkeland[1], Vandana Ardawatia[1], Ingeborg Winge[1], Sura Aziz[1,2], Gøril Knutsvik[1,2], Elisabeth Wik[1,2], Joao A. Paulo[3], Heidrun Vethe[1], Dimitrios Kleftogiannis[1,4], Lars A. Akslen[1,2*].

[1] Centre for Cancer Biomarkers CCBIO, Department of Clinical Medicine, Section for Pathology, University of Bergen, Bergen N-5021, Norway

[2] Department of Pathology, Haukeland University Hospital, Bergen N-5021, Norway

[3] Department of Cell Biology, Harvard Medical School, Boston, MA, USA

[4] Department of Informatics, Computational Biology Unit, University of Bergen, Bergen, Norway

*Corresponding author (E-mail: lars.akslen@uib.no)

Supplementary Figures

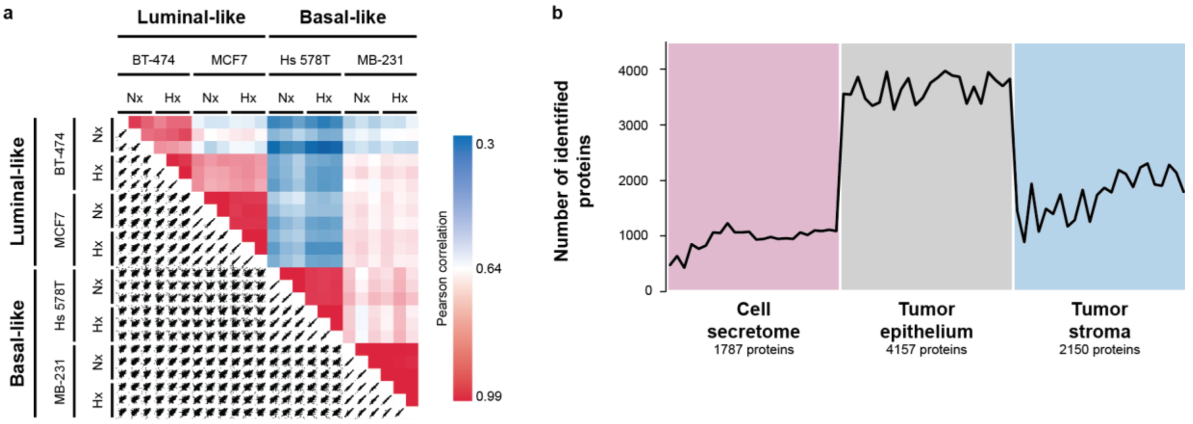

Supplementary Figure 1: Mass spectrometry data of technical replicates and number of identified proteins.

Matrix of cell secretome sample correlation plots of discovery BCCL panel (n=4) **(a)** shows high correlation between technical replicates (0.85-0.99), suggesting good data quality. Color represents value of the Pearson correlation coefficient. High correlation was found between oxygen conditions within the cell lines (lowest Pearson correlation coefficient 0.77 in BT474), and a greater variation between cell lines of different subtypes (0.30-0.70). Plot of number of identified proteins in each sample **(b)**. 1,787 proteins were identified in breast cancer cell secretome samples of discovery BCCLs, 4,157 proteins detected in microdissected tumor epithelium, and 2,150 proteins detected in microdissected tumor stroma.

BCCL: Breast cancer cell line. FFPE: Formalin-fixed paraffin-embedded tissue. Hx: Hypoxia. Nx: Normoxia.

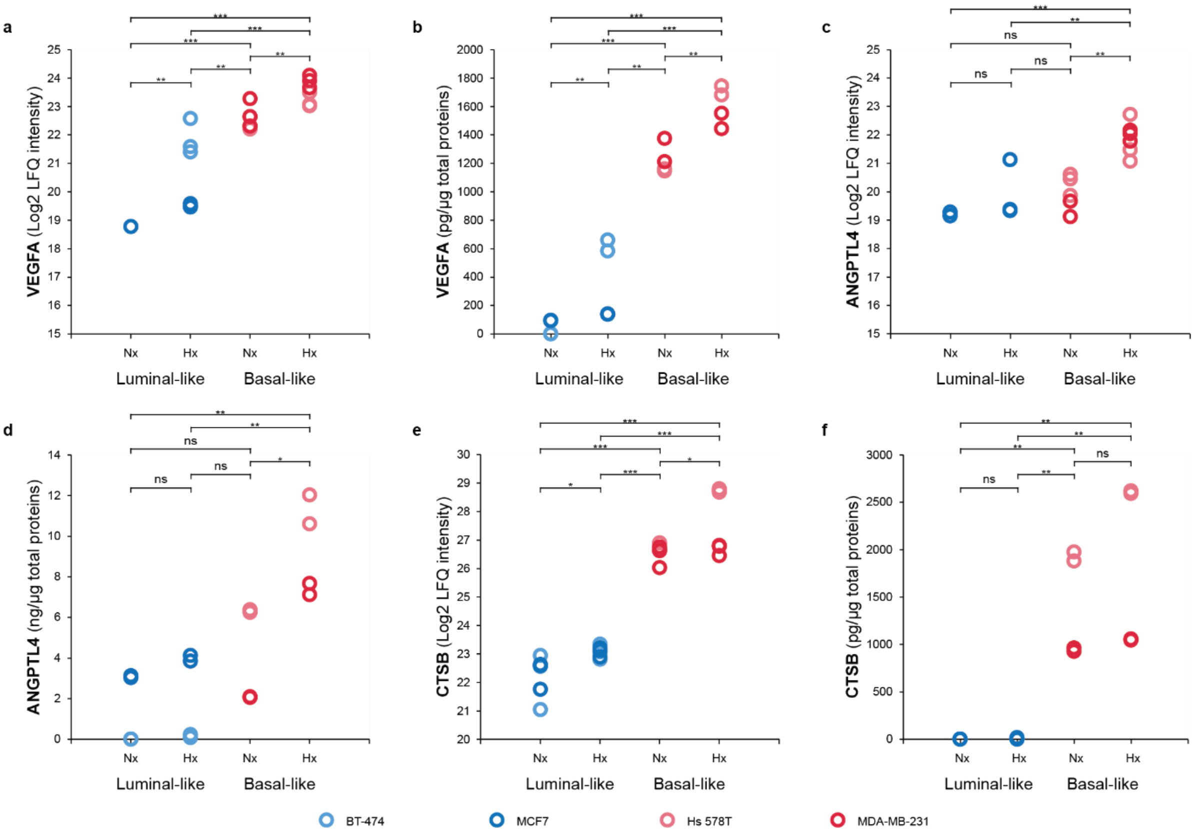

Supplementary Figure 2: Relative abundance of VEGFA, ANGPTL4 and CTSB from MS analysis and validation by ELISA.

Plot of LFQ intensity detected in MS analysis for VEGFA **(a)**, ANGPTL4 **(c)**, and CTSB **(e)** and validation with ELISA **(b, d, f)**. Plots show consistent patterns between MS analysis and ELISA results, with significant hypoxia-increased secretion of VEGFA, ANGPTL4 and CTSB in basal-like cell lines (red), as well as VEGFA in luminal-like cell lines (blue). CTSB was not detected by ELISA in luminal-like secretomes, however, consistent with lower levels of secretion from luminal-like than basal-like cell lines. CTSB showed significantly increased secretion in response to hypoxia in basal-like cell lines when inspecting the individual cell lines (Hs 578T and MDA-MB-231). LFQ intensities are derived from triplicates of each cell line (n=4), and ELISA measurements are derived from duplicate readings of independent samples (n=4). The data were grouped for each subtype (luminal-like or basal-like).
Source data are provided as a Source Data file.

*	Two-sided Student's T-test, significance level 0.05.
**	Two-sided Student's T-test, significance level 0.01.
***	Two-sided Student's T-test, significance level 0.001.

ANGPTL4: Angiopoietin-like 4. CTSB: Cathepsin B. ELISA: Enzyme-linked immunosorbent assay. Hx: hypoxia. LFQ: Label-free quantification. MS: Mass spectrometry. Nx: normoxia. VEGFA: Vascular endothelial growth factor A.

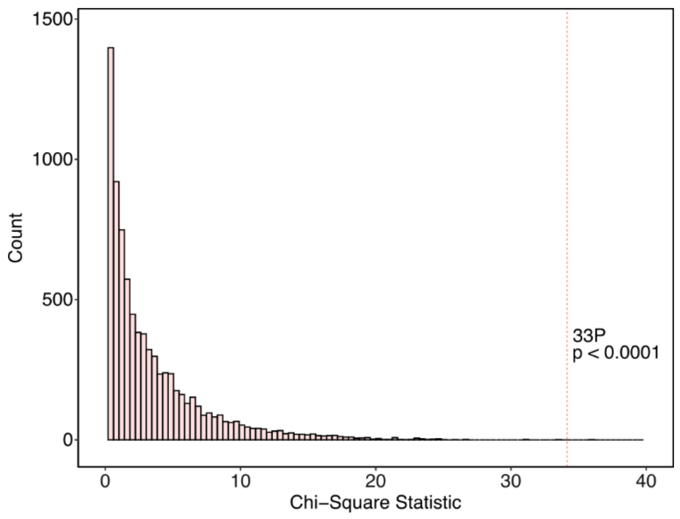

Supplementary Figure 3: Permutation test.

Histogram of cumulative chi-square statistics values after 10,000 permutations. In each permutation, 33 proteins were selected at random from a pool of the 150 hypoxia-increased and 283 stroma proteins from which the 33P was derived, and the one-sided chi-square statistics from a univariate survival analysis (Kaplan-Meier method) were extracted. The dotted red line shows the chi-square statistics of the 33P signature. The p-value is calculated from the proportion of permutations that give a higher Chi-Square value divided by the total number of permutations.

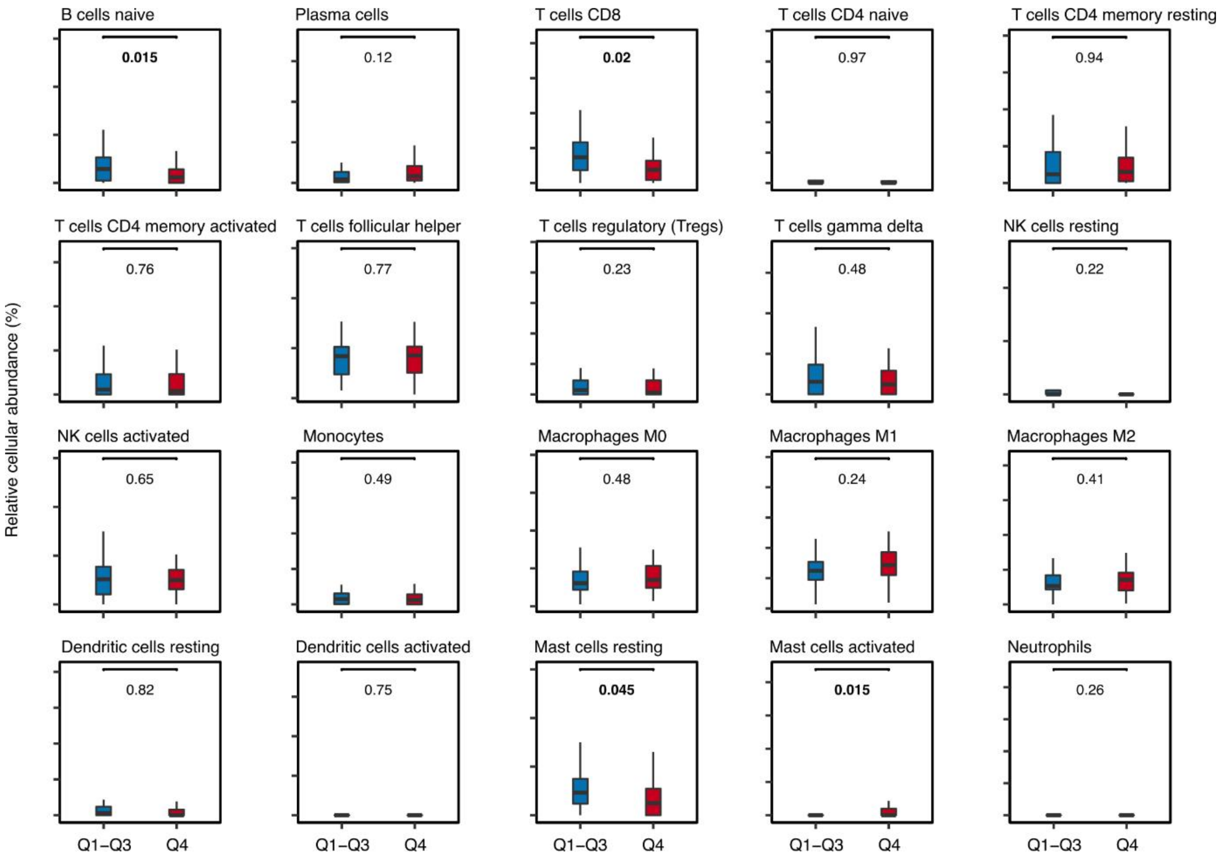

Supplementary Figure 4: Cibersort cell deconvolution analysis of basal-like patients in the METABRIC-Discovery cohort.

The Cibersort cell deconvolution tool was used to examine potential associations between 33P and specific cell types in the tumor stroma of basal-like breast cancer. The relative cellular abundance for each cell type was compared between 33P high (Q4, poor survival, n = 73) versus the rest (Q1-Q3, better survival, n = 45. B-cells, CD8 T-cells and resting mast cells show lower relative abundance in the 33P high group, while activated mast cells show higher cellular abundance in the 33P high group compared with the rest (Q1-Q3). The boxplots display the median (represented by the center bar) and the third and first quartiles (shown as the upper and lower edges, respectively). The whiskers of the boxplot extend to the most extreme data point within a range that is no more than 1.5 times the interquartile range from the box. Potential outliers are not shown.
Statistical test: Two-sided Wilcoxon signed-rank test. Adjusting for testing multiple cell types was not performed.

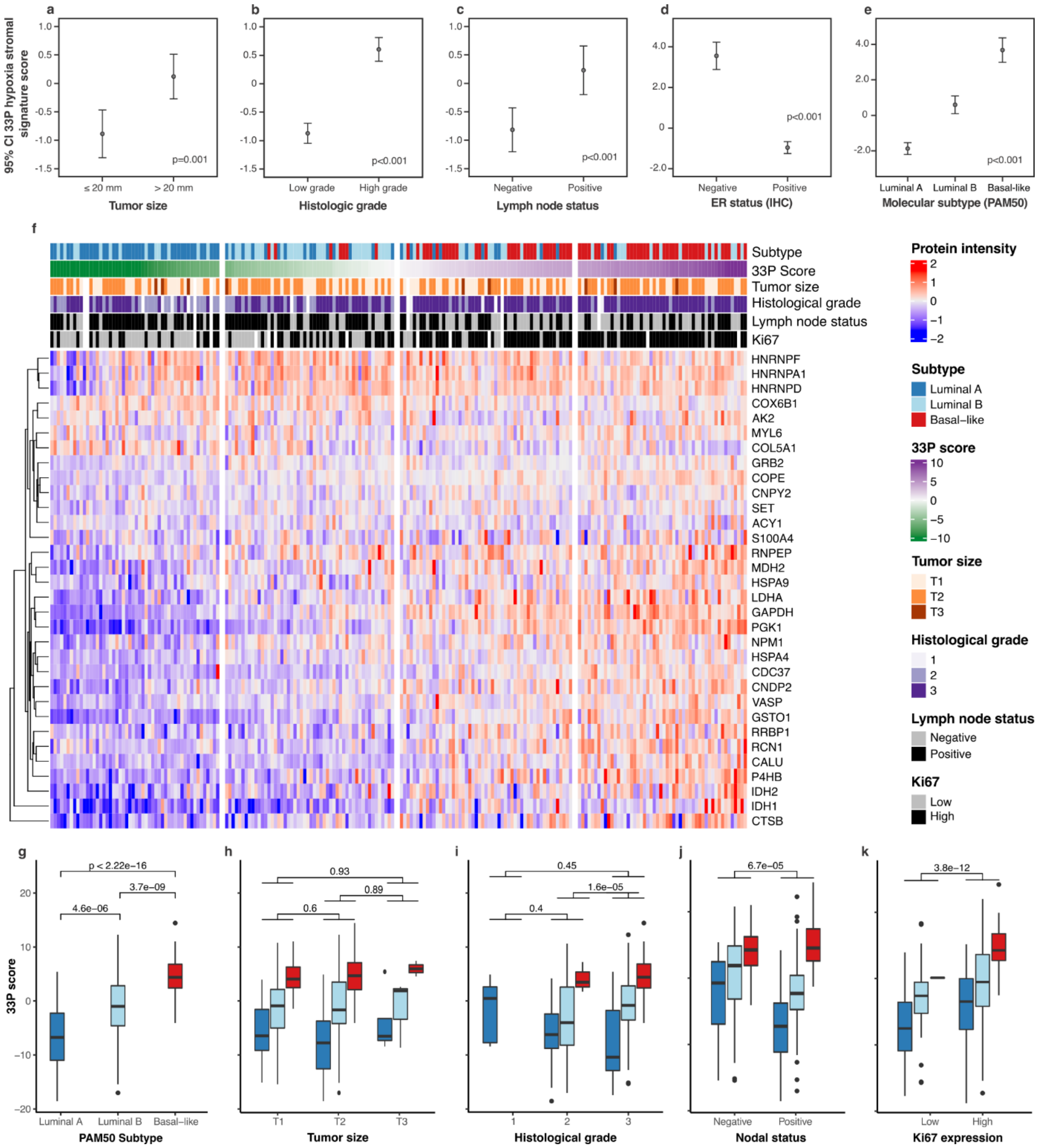

Supplementary Figure 5: Exploration of 33P and clinico-pathological features in METABRIC-Discovery (transcriptomics) and Asleh *et al*. (2022) (proteomics).

Error bars representing 95% confidence interval of the mean and show that high 33P mRNA score associates with large tumor size **(a)**, high histologic grade **(b)**, lymph node metastases **(c)**, ER-negative tumors **(d)**, and the basal-like subtype (PAM50) **(e)** in the METABRIC-Discovery cohort. Heatmap of the 33P hypoxia signature protein expression in luminal A, luminal B and basal-like patients (n=209) included in the Asleh *et al*. (2022) study **(f)**. Patients are sorted supervised from left to right with increasing 33P signature score. The rows are clustered unsupervised (distance: Euclidean, method: complete). **(g-k)** Each of the annotated variables PAM50 subtype **(g)**, tumor size **(h)**, histological grade **(i)**, lymph node status **(j)** and KI67 expression **(k)** was plotted (boxplot) separately against 33P score and stratified into PAM50 subgroups (luminal A, n=66; luminal B, n=70; basal-like, n=73). The boxplots show significant associations between high 33P signature scores and PAM50 subtype, histologic grade (grade 1-2 versus grade 3), lymph node status and Ki67 expression. The boxplots display the mean (represented by the center bar) and the third and first quartiles (shown as the upper and lower edges, respectively). The whiskers of the boxplot extend to the most extreme data point within a range that is no more than 1.5 times the interquartile range from the box. The data points above and below the whiskers are potential outliers.

Statistical test: Two-sided Mann-Whitney U-test. Adjustment for multiple testing was not performed.
CI: confidence interval; ER: estrogen receptor; IHC: immunohistochemistry.

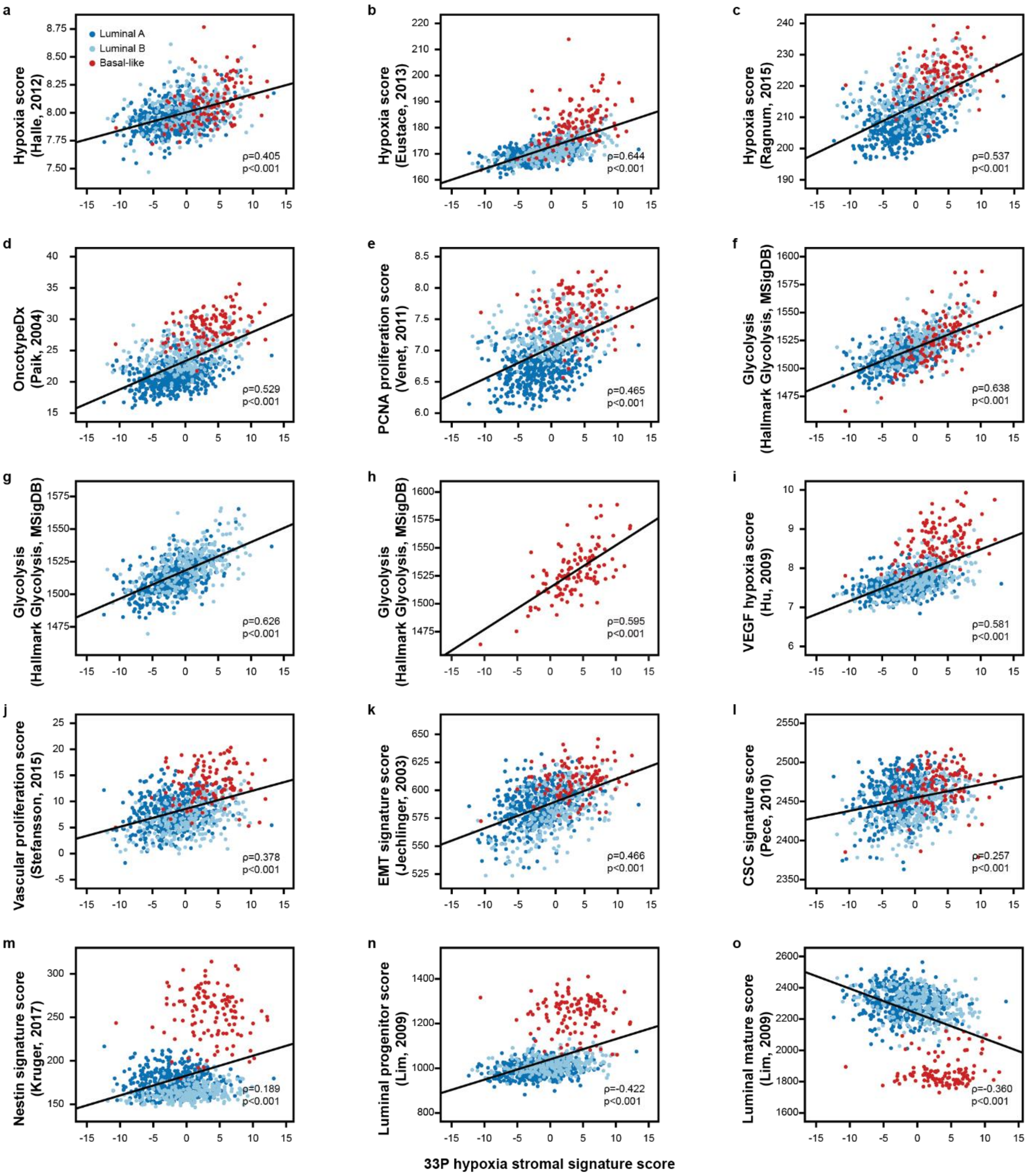

Supplementary Figure 6: Signature correlations.

33P hypoxia stromal signature correlates with signatures for tissue hypoxia **(a-c)**, proliferation **(d-e)**, glycolysis **(f-h)**, vascular proliferation **(i-j)**, and signatures reflecting EMT **(k)** and stemness **(l-m)**, a luminal progenitor signature **(n)** and correlates negatively with mature luminal signature **(o)** in the METABRIC-Discovery mRNA cohort.
ρ: Spearman's rank correlation coefficient.
p: Spearman's rho test (two-sided)

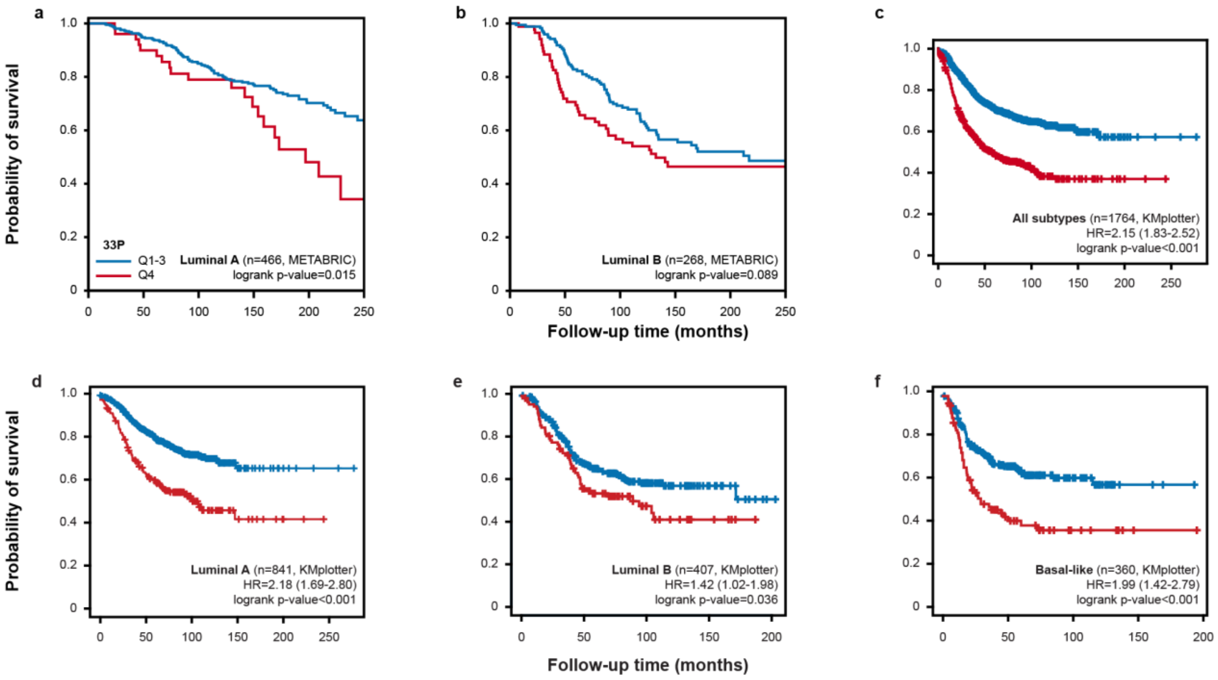

Supplementary Figure 7: Survival plots in breast cancer patients scored by 33P hypoxia stromal signature.

Kaplan-Meier plots of luminal A (n=466) **(a)** and luminal B (n=268) **(b)** from the METABRIC-Discovery mRNA cohort. The plots show a significant association between high signature scores and poor survival for luminal-like A subtype. Validation in the merged cohorts from KMplotter (updated n=4934) **(c)**, and also stratified for luminal A **(d)**, luminal B **(e)**, and basal-like subtype **(f)**, show significantly lower probability of survival of patients with high 33P scores. Red lines represent the 33P high (upper quartile, Q4) group, and the blue line represents the rest (Q1- Q3). Survival differences between groups were evaluated with a two-sided log-rank test.

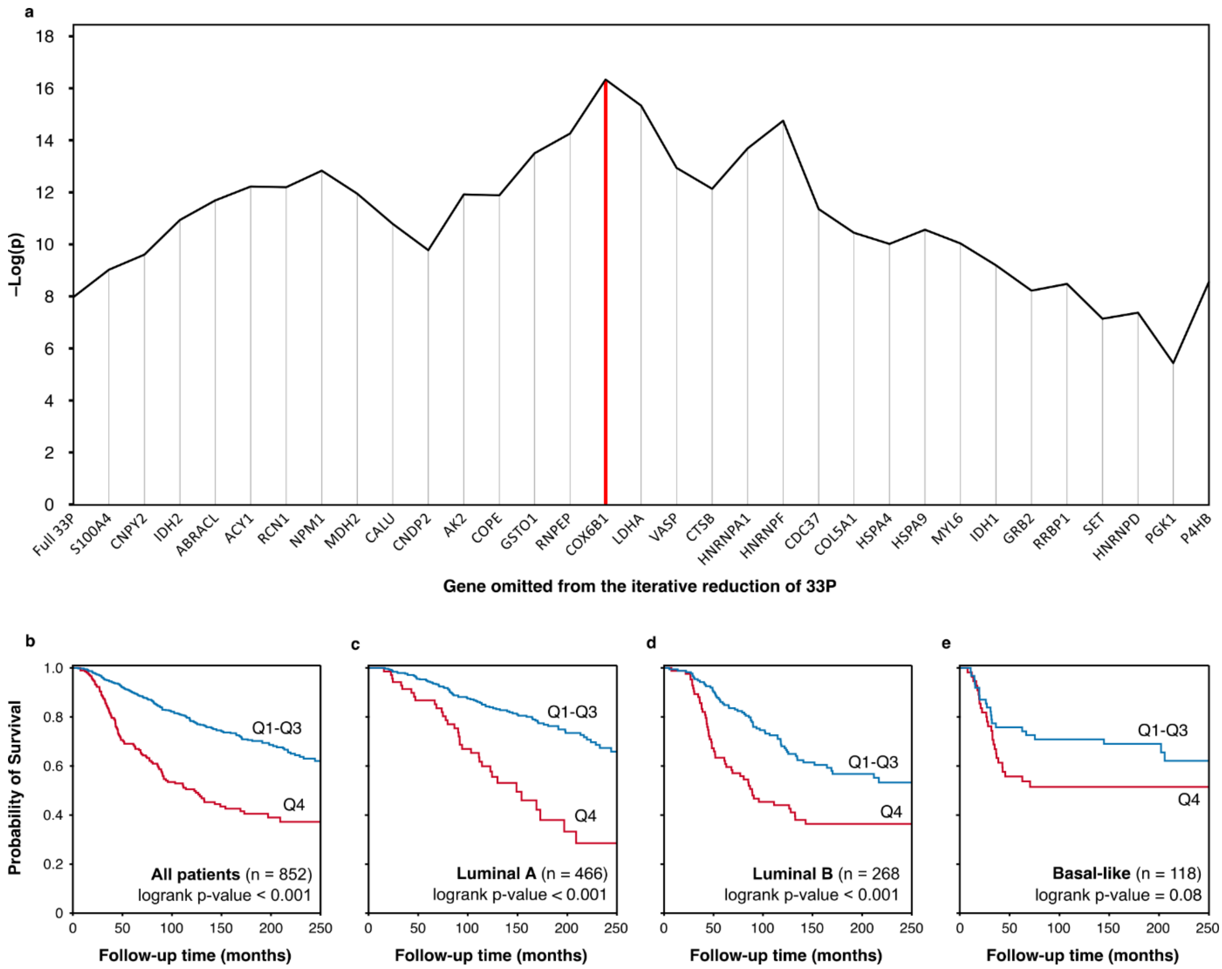

Supplementary Figure 8: Reduction analysis of 33P identifies a 'peak signature' of 18 proteins.

The 33P signature was reduced by recursively leaving one gene out of the signature and then testing the predictive strength of the remaining N-1 genes in a survival analysis (Q1-3 vs Q4, METABRIC-Discovery cohort, n=852). The strongest N-1 signature (lowest Log-rank p-value) was retained, and the process was repeated until only one gene remained**. (a)** The mountain-like plot shows the log-rank p-value for each iteration. The red line represents the "peak-signature", i.e., the reduced version of 33P (18-proteins) that showed the largest effect on survival (p= 4.3 x 10-17, compared to baseline 33P p=1.0 10-8). The 18-proteins were CDC37, COL5A1, CTSB, GAPDH, GRB2, HNRNPA1, HNRNPD, HNRNPF, HSPA4, HSPA9, IDH1, LDHA, MYL6, P4HB, PGK1, RRBP1, SET and VASP. **(b-e)** Univariate survival analysis (Kaplan-Meier method) of the 18-protein "peak"-signature in all patients (n = 852), Luminal A (n = 466), Luminal B (n = 268) and basal-like (n = 118) in the METABRIC-Discovery cohort. The reduction analysis was based on "all patients". Still, after stratifying the patients we observed a lower survival in Q4 of both the luminal A and luminal B subtypes compared to the original 33P-signature. Red lines represent the 33P high (upper quartile, Q4) group, and the blue line represents the rest (Q1- Q3). Survival differences between groups were evaluated with a two-sided log-rank test.

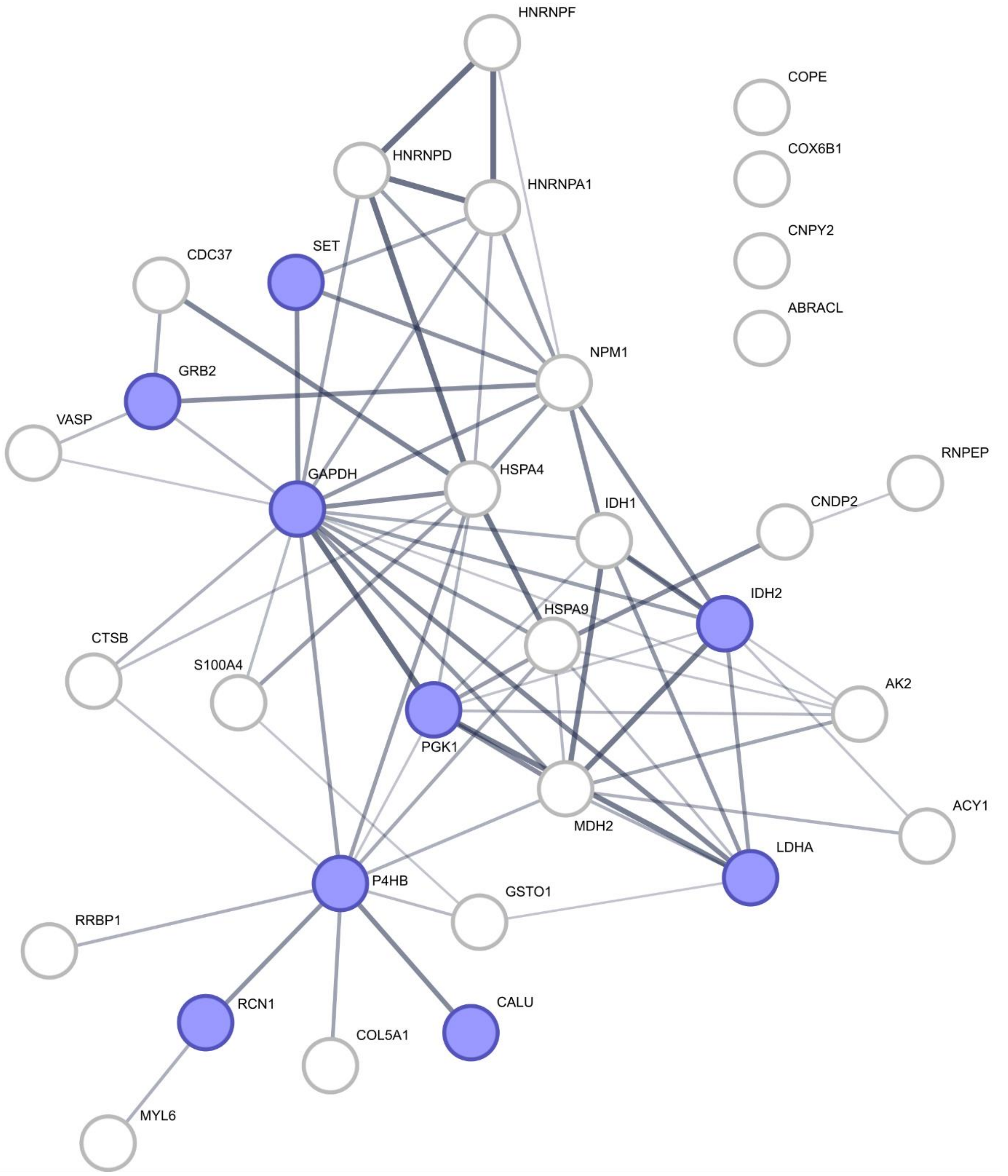

Supplementary Figure 9: Protein-protein association network of the 33P proteins.

The protein-protein association network shows that 29 of the 33 proteins are connected to one large network. Thickness of lines between proteins represents strength of association. The blue colored nodes are associated with "VEGFA-VEGFR2 signaling pathway" (WikiPathways; WP3888; p<0.001). The figure was generated from string-db.org.

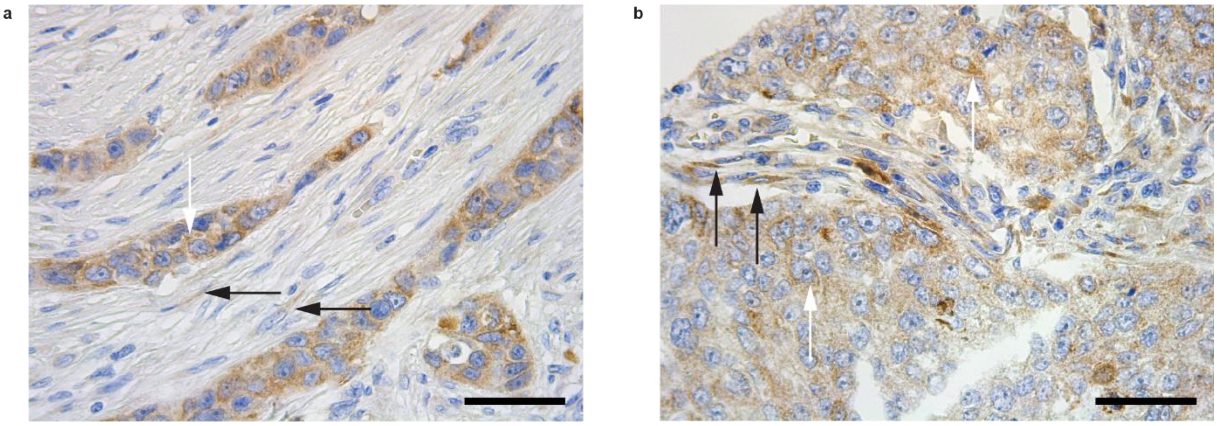

Supplementary Figure 10: Immunohistochemical staining of NRF2 in breast cancer tissues.

Evaluation of NRF2 expression in breast cancer tissue microarrays by immunohistochemistry (n=42; x400 magnification, scale bar 50 µm), showing weak **(a)** and moderate **(b)** stromal staining. Stromal expression is indicated with black arrows, and tumor epithelial staining is indicated with white arrows. Stronger stromal expression of NRF2 is positively correlated with 33P scores (MS-proteomics) in the same samples (p=0.05), but tumor cell expression is not associated with 33P.

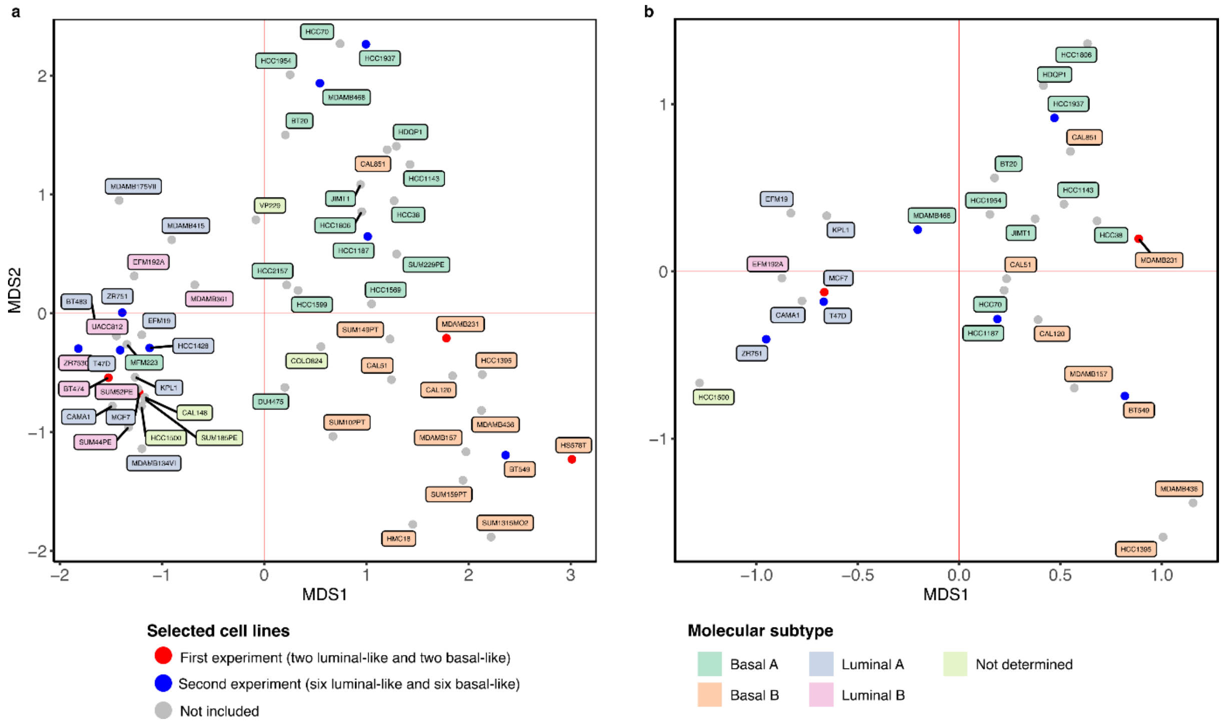

Supplementary Figure 11: Multi-dimensional scaling plots of gene and protein expression in breast cancer cell lines from the Cancer Cell Line Encyclopedia.

Multi-dimensional scaling plot of the global transcriptomic **(a)** and proteomic **(b)** data from breast cancer cell lines in the Cancer Cell Line Encyclopedia (CCLE). Cell lines that were included in the first (discovery) experiment are shown as red dots. Cell lines that were added in the validation dataset are shown as a blue dot. The CCLE transcriptomic and proteomic data was used to ensure a representative selection of cell lines. Of note, the CCLE database did not include proteomic data for all breast cancer cell lines from **(a)**, including four of the cell lines included in our studies.

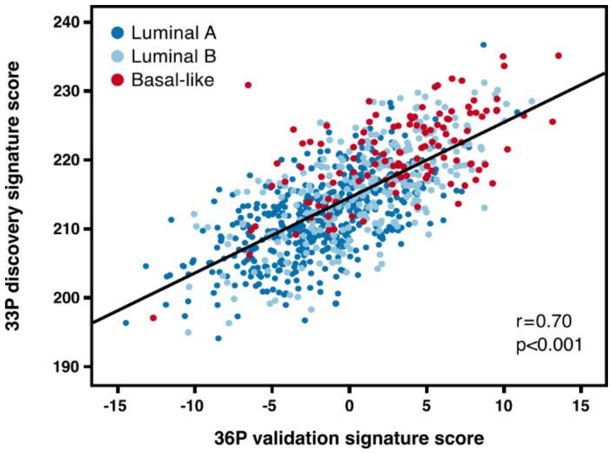

Supplementary Figure 12: Scatterplot of the 33P discovery signature score against the 36P validation signature score.

Each dot represents one patient in the METABRIC-Discovery cohort (n=852; luminal-like and basal-like only). A Pearson correlation coefficient (r) of 0.70 suggests a strong correlation between the discovery and validation signatures. Statistical test: two-tailed t-test.

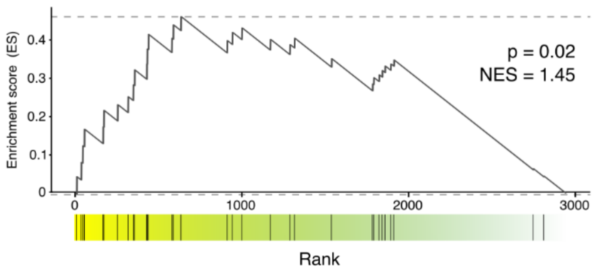

Supplementary Figure 13: Gene set enrichment analysis of 33P in the hypoxia validation dataset.

The identified proteins in the validation dataset were ranked by p-value (all samples, paired t-test (two-sided), no adjustment was performed since only one gene set was tested – hypoxia vs. normoxia) and tested against the 33P proteins in a gene set enrichment analysis. The analysis showed a significant enrichment of 33P in the hypoxia validation dataset (p=0.02; NES 1.45). The figure was generated using the fgsea R-package.

ES: enrichment score; NES: normalized enrichment score.

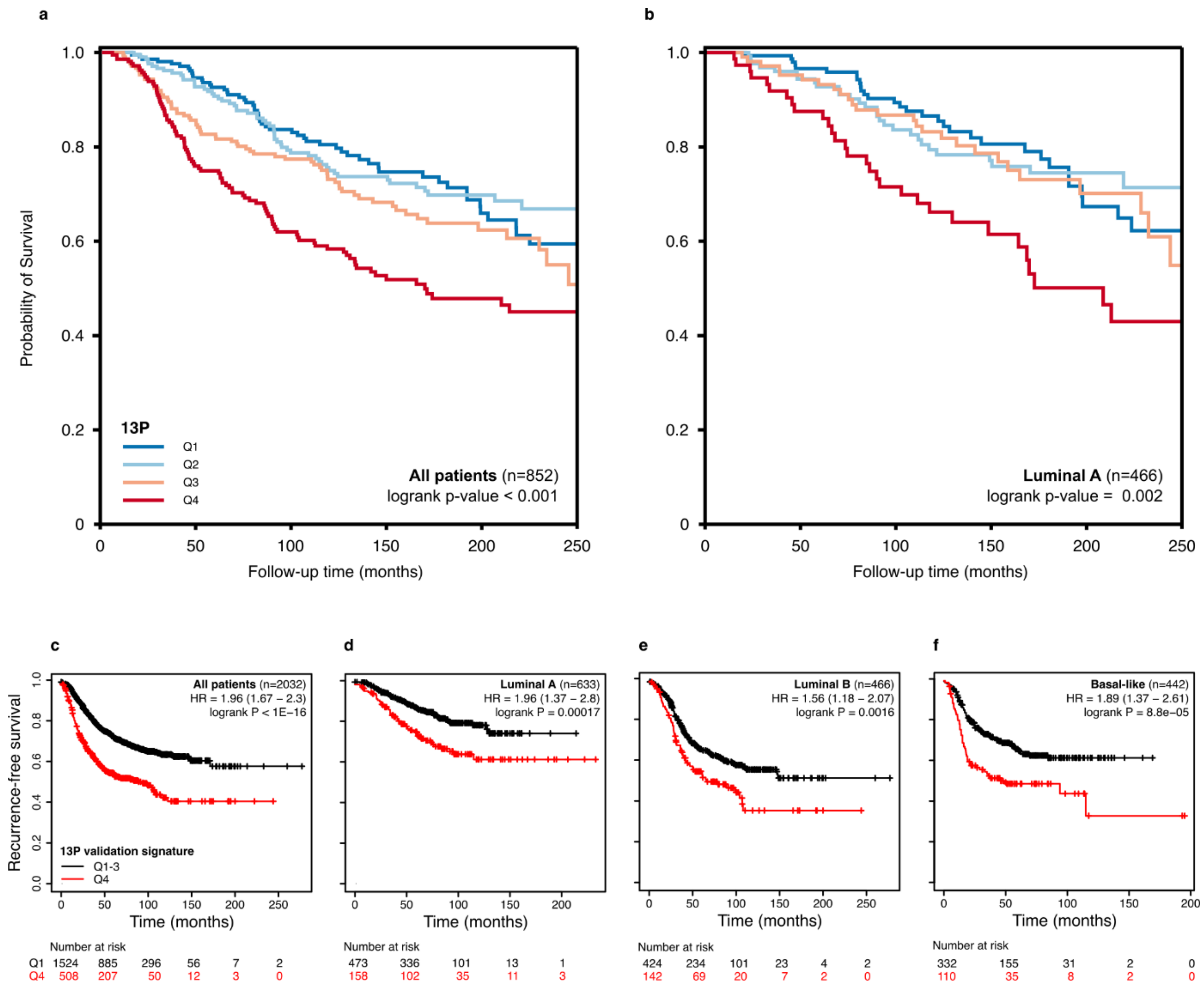

Supplementary Figure 14: Univariate survival analysis (Kaplan-Meier method) of patients from METABRIC-Discovery cohort and KMplotter by expression of the 13P genes.

Patients in the METABRIC-Discovery cohort were grouped into four quartiles (Q1-Q4) based on the expression of the 13P genes, and both **(a)** all patients and **(b)** the patients diagnosed with luminal A breast cancer showed worse probability of survival in the high 13P group. These data were supported by KMplotter, where high 13P (upper quartile) was associated with worse survival in **(c)** all patients (n=2032), **(d)** luminal A (n=633), **(e)** luminal B (n=466) and **(f)** basal-like patients (n=442). Survival differences between groups were evaluated with a two-sided log-rank test.

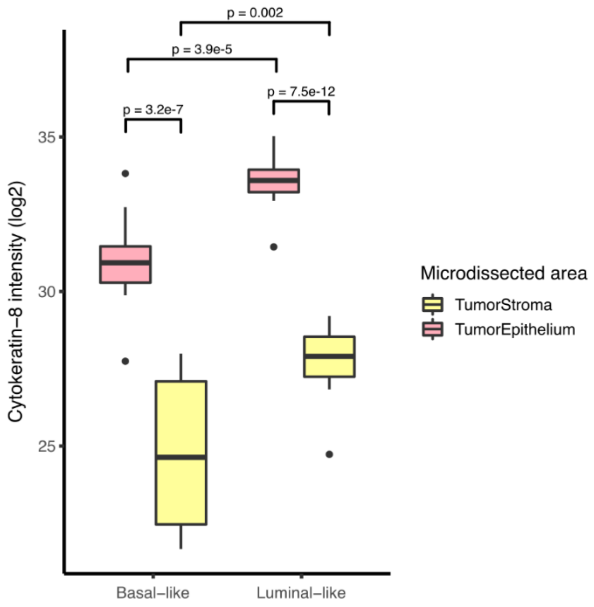

Supplementary Figure 15: Cytokeratin-8 protein expression in microdissected tumor stroma and tumor epithelium.

Boxplots showing the expression of cytoketain-8 in basal-like (n = 12) and luminal-like (n = 12) tumor stroma and epithelium. Cytokeratin-8 is an epithelial marker and is not expected to be expressed in tumor stroma. Therefore, this marker was used to estimate the amount of tumor epithelial cells in the microdissected stromal fraction. The amount of cytokeratin was, on average, 62-fold higher in tumor epithelium compared with tumor stroma (basal-like: 68-fold, p=3.2e-7; luminal-like: 56-fold, p=7.5e-12; two-sided Student's t-test), which translates to an average contamination of only 1.6% epithelium in the stromal fractions. The low levels of epithelium in microdissected stroma were true for both basal-like and luminal-like samples. The boxplots display the mean (represented by the center bar) and the third and first quartiles (shown as the upper and lower edges, respectively). The whiskers of the boxplot extend to the most extreme data point within a range that is no more than 1.5 times the interquartile range from the box. The data points above and below the whiskers are potential outliers.

Supplementary tables

Supplementary Table 1: Selected cell lines.

Cell line	Subtype	Receptor status			Tumor type	Source	Literature
		ER	PR	HER2			
**Initial selection (discovery panel)**							
**BT-474**	Luminal (B)	+	+	+	IDC	PT	[1-5]
**MCF 7**	Luminal (A)	+	+	-	IDC	PE	[1-5]
**Hs 578T**	Basal B (claudin-low)	-	-	-	IDC	PT	[1-5]
**MDA-MB-231**	Basal B (claudin-low)	-	-	-	AC	PE	[1-5]
**Additionally selected cell lines (validation panel)**							
**HCC1428**	Luminal (A)	+	+	-	AC	PE	[1-3,5]
**T47D**	Luminal (A)	+	+	-	IDC	PE	[1-5]
**ZR751**	Luminal (A)	+	-	-	IDC	AF	[1-3,5]
**ZR-75-30**	Luminal (B)	+	-	+	IDC	AF	[1-3,5]
**MDA-MB-468**	Basal A	-	-	-	AC	PE	[1-5]
**HCC1143**	Basal A	-	-	-	DC	PT	[1-3,5]
**HCC1187**	Basal A	-	-	-	DC	PT	[1-3,5]
**BT-549**	Basal B (claudin-low)	-	-	-	IDC	PT	[1,2,5]

AC: adenocarcinoma. AF: ascites fluid. DC: ductal carcinoma. ER: estrogen receptor. HER2: human epidermal growth factor receptor 2. IDC: invasive ductal carcinoma. PE: pleural effusion. PR: progesterone receptor. PT: primary tumor. For additional information, see Neve *et al.*[1], Dai *et al.*[2], Kao *et al.*[3], Holliday *et al.*[4], and Nusinow *et al.*[5].

Supplementary Table 2: Subclusters of hypoxia-upregulated metabolic processes.

Gene name	Protein name	Subcluster
ACO1 [(1)]	Cytoplasmic aconitate hydratase	**Subcluster 1:**<br>10 nodes, 36 edges<br><br>GOBP*: Tricarboxylic acid cycle [(1)]
FH [(1)]	Fumarate hydratase, mitochondrial	
FBP1	Fructose-1,6-bisphosphatase 1	
GOT1	Aspartate aminotransferase, cytoplasmic	
GPI	Glucose-6-phosphate isomerase	
IDH1 [(1)]	Isocitrate dehydrogenase [NADP] cytoplasmic	
IDH2 [(1)]	Isocitrate dehydrogenase [NADP], mitochondrial	
MDH1 [(1)]	Malate dehydrogenase, cytoplasmic	
ME1	NADP-dependent malic enzyme	
TXNRD1	Thioredoxin reductase 1, cytoplasmic	
CYCS	Cytochrome c	**Subcluster 2:**<br>15 nodes, 52 edges<br><br>GOBP*: Negative regulation of apoptosis, glycolysis [(1)]
GAPDH [(1)]	Glyceraldehyde-3-phosphate dehydrogenase	
HSPA4	Heat shock 70 kDa protein 4	
HSPA9	Stress-70 protein, mitochondrial	
HSPH1	Heat shock protein 105 kDa	
LDHA [(1)]	L-lactate dehydrogenase A chain	
MDH2 [(1)]	Malate dehydrogenase, mitochondrial	
NME1	Nucleoside diphosphate kinase A	
NME2	Nucleoside diphosphate kinase B	
NPM1	Nucleophosmin	
PRDX4	Peroxiredoxin-4	
PRDX5	Peroxiredoxin-5, mitochondrial	
SUCLG2	Succinyl-CoA ligase [GDP-forming] subunit beta, mitochondrial	
TXNDC5	Thioredoxin domain-containing protein 5	
VEGFA	Vascular endothelial growth factor A	
AK2	Adenylate kinase 2, mitochondrial	**Subcluster 3:**<br>7 nodes, 12 edges<br><br>GOBP*: Membrane to membrane docking, cell redox homeostasis [(1)]
EZR	Ezrin	
HSPE1	10 kDa heat shock protein, mitochondrial	
MSN	Moesin	
P4HB [(1)]	Protein disulfide-isomerase	
PGK1	Phosphoglycerate kinase 1	
TXN [(1)]	Thioredoxin	

GOBP: Gene ontology biological process.

* Biological process significantly overrepresented in subcluster, PANTHER Overrepresentation Test (Released 2018-11-13), GO Ontology database released 2019-01-01. Statistical test: One-sided Fisher's exact test. Adjusting for multiple testing was performed using the Benjamini-Hochberg false discovery rate (FDR) method.

[(1)] Protein involved in marked enriched biological process for subcluster.

Supplementary Table 3: 33P hypoxia stromal signature.

Protein IDs	Gene name	Stromal subtype	Fold change [(1)]
Q03154	ACY1	Luminal-like	2.39
P20908	COL5A1 [(2)]	Luminal-like	1.97
Q9H4A4	RNPEP [(2)]	Luminal-like	1.29
Q9P1F3	ABRACL	Basal-like	1.80
P54819	AK2 [(2)]	Basal-like	1.73
O43852	CALU	Basal-like	1.69
Q16543	CDC37	Basal-like	1.53
Q96KP4	CNDP2	Basal-like	1.42
Q9Y2B0	CNPY2	Basal-like	2.45
O14579	COPE	Basal-like	2.51
P14854	COX6B1	Basal-like	2.23
P07858	CTSB	Basal-like	2.89
P04406	GAPDH [(2)]	Basal-like	1.71
P62993	GRB2	Basal-like	2.22
P78417	GSTO1 [(2)]	Basal-like	1.32
P09651	HNRNPA1	Basal-like	1.99
Q14103	HNRNPD	Basal-like	1.62
P52597	HNRNPF [(2)]	Basal-like	1.68
P34932	HSPA4 [(2)]	Basal-like	1.83
P38646	HSPA9	Basal-like	1.46
O75874	IDH1 [(2)]	Basal-like	4.86
P48735	IDH2	Basal-like	2.71
P00338	LDHA [(2)]	Basal-like	1.71
P40926	MDH2	Basal-like	2.00
P60660	MYL6	Basal-like	1.37
P06748	NPM1 [(2)]	Basal-like	1.88
P07237	P4HB	Basal-like	1.62
P00558	PGK1 [(2)]	Basal-like	1.77
Q15293	RCN1	Basal-like	1.34
Q9P2E9	RRBP1	Basal-like	1.72
P26447	S100A4	Basal-like	1.59
Q01105	SET [(2)]	Basal-like	1.79
P50552	VASP [(2)]	Basal-like	1.89

(1) Fold change between luminal-like and basal-like subtype in microdissected stromal samples.
(2) Proteins in 13-protein subsignature of 33P.

Supplementary Table 4: Proteins in common for differentially secreted or expressed proteins with signatures for breast cancer subtypes, hypoxia, and stromal features.

**Oxygen conditions/hypoxia** [(1)]		
	**Overlapping genes/proteins**	**Signature/gene set**
**Breast cancer hypoxia response proteins** (150 proteins)	GAPDH, AK2	Halle, 2012 (31 genes)
	VEGFA, ANGPTL4, LDHA, PGK1	Eustace, 2013 (26 genes)
	RNASE4	Ragnum, 2015 (32 genes)
**Stromal hypoxia** [(2)]		
	**Overlapping genes/proteins**	**Signature/gene set**
**33P stromal-based hypoxia signature** (33 proteins)	**Hypoxia signatures**	
	AK2, GAPDH	Halle, 2012 (31 genes)
	LDHA, PGK1	Eustace, 2013 (26 genes)
	–	Ragnum, 2015 (32 genes)
	**Proliferation signatures**	
	GAPDH	OncotypeDx; Paik, 2004 (21 genes)
	–	PCNA proliferation signature; Venet, 2011 (131 genes)
	**Glycolysis**	
	COL5A1, MDH2, LDHA, IDH1, PGK1	Hallmark glycolysis (200 genes)
	**Vascular proliferation**	
	–	Hu, 2009 (13 genes)
	–	Stefansson, 2015 (32 genes)
	**EMT and stemness**	
	–	Jechlinger, 2003 (128 genes)
	P4HB, GAPDH, AK2	Pece, 2010 (299 genes)
	–	Kruger, 2017 (44 genes)
	CTSB	Luminal progenitor signature; Lim, 2009 (626 genes)
	AK2, HNRNPA1	Mature luminal signature; Lim, 2009 (990 genes)

(1) Oxygen conditions/hypoxia: breast cancer hypoxia response proteins (150 proteins) consist of proteins with increased secretion in response to hypoxia; proteins with significantly higher secretion from hypoxic vs. normoxic breast cancer cell line secretomes. Two-sided Student's t-test, significance level $p<0.05$.

(2) Stromal hypoxia: 33P stromal-based hypoxia signature (33 proteins) derived from breast cancer hypoxia response proteins and stromal proteome information.

Supplementary Table 5: Multivariate survival analysis (proportional hazards regression model) stratified by molecular subtype.

Variable	n	Univariate analysis		Multivariate analysis	
		HR (95 % CI)	p-value	HR (95 % CI)	p-value
**Luminal-like subtype** (n=734)					
**Tumor size**					
≤ 20 mm	228	1.00	<0.0005	1.00	<0.0005
> 20 mm	506	2.26 (1.64-3.11)		1.89 (1.36-2.61)	
**Histologic grade**					
1-2	435	1.00	<0.0005	1.00	0.005
3	299	1.80 (1.40-2.33)		1.46 (1.12-1.90)	
**Lymph node status**					
Negative	400	1.00	<0.0005	1.00	<0.0005
Positive	334	2.02 (1.56-2.63)		1.68 (1.29-2.20)	
**33P hypoxia stromal signature**					
Q123	594	1.00	<0.0005	1.00	0.003
Q4	140	1.84 (1.38-2.46)		1.57 (1.17-2.11)	
**Basal-like subtype** (n=118)					
**Tumor size**					
≤ 20 mm	35	1.00	NS	1.00	NS
> 20 mm	83	0.90 (0.49-1.68)		0.70 (0.37-1.31)	
**Histologic grade**					
1-2	8	1.00	NS	1.00	NS
3	110	2.04 (0.47-8.42)		1.47 (0.35-6.19)	
**Lymph node status**					
Negative	53	1.00	0.006	1.00	0.014
Positive	65	2.39 (1.28-4.48)		2.26 (1.18-4.33)	
**33P hypoxia stromal signature**					
Q123	45	1.00	0.014	1.00	0.030
Q4	73	2.28 (1.18-4.40)		2.10 (1.08-4.08)	

CI: confidence interval. HR: hazard ratio. n: number of patients. NS: not significant.
Statistical test: Two-sided Wald test. Adjustment for multiple testing was not performed.

Supplementary references

1 Neve, R. M. *et al.* A collection of breast cancer cell lines for the study of functionally distinct cancer subtypes. *Cancer Cell* **10**, 515-527 (2006). https://doi.org:10.1016/j.ccr.2006.10.008

2 Dai, X., Cheng, H., Bai, Z. & Li, J. Breast Cancer Cell Line Classification and Its Relevance with Breast Tumor Subtyping. *J Cancer* **8**, 3131-3141 (2017). https://doi.org:10.7150/jca.18457

3 Kao, J. *et al.* Molecular profiling of breast cancer cell lines defines relevant tumor models and provides a resource for cancer gene discovery. *PLoS One* **4**, e6146 (2009). https://doi.org:10.1371/journal.pone.0006146

4 Holliday, D. L. & Speirs, V. Choosing the right cell line for breast cancer research. *Breast Cancer Res* **13**, 215 (2011). https://doi.org:10.1186/bcr2889

5 Nusinow, D. P. *et al.* Quantitative Proteomics of the Cancer Cell Line Encyclopedia. *Cell* **180**, 387-402 e316 (2020). https://doi.org:10.1016/j.cell.2019.12.023
