# Supplementary material for: Hypoxia induced responses are reflected in the stromal proteome of breast cancer
Source: Nat Commun. 2023 Jun 22;14:3724. doi: 10.1038/s41467-023-39287-7 (PMC10287711; doi:10.1038/s41467-023-39287-7)
Supplement: Supplementary file 3 — Description of Additional Supplementary Files [file 41467_2023_39287_MOESM3_ESM.pdf]

Description of Additional Supplementary Files

File Name: Supplementary Data 1
Description: Significantly enriched biological processes at baseline (normoxia).

File Name: Supplementary Data 2
Description: Proteins significantly upregulated in response to hypoxia in breast cancer cell lines.

File Name: Supplementary Data 3
Description: Top 5 upstream transcriptional regulators of the hypoxia-upregulated proteins.

File Name: Supplementary Data 4
Description: Hypoxia-upregulated biological processes in main network (125 of 150 hypoxia-upregulated proteins).

File Name: Supplementary Data 5
Description: Hypoxia-upregulated biological processes, luminal-like secretome.

File Name: Supplementary Data 6
Description: Hypoxia-upregulated biological processes, basal-like secretome.

File Name: Supplementary Data 7
Description: Drug signatures negatively correlated to high 33P.

File Name: Supplementary Data 8
Description: Multivariate survival analysis (proportional hazards regression model) including 13P hypoxia validation signature in breast cancer patients (METABRIC-Discovery cohort, n=832).
