# Supplementary material for: Hypoxia induced responses are reflected in the stromal proteome of breast cancer
Source: Nat Commun. 2023 Jun 22;14:3724. doi: 10.1038/s41467-023-39287-7 (PMC10287711; doi:10.1038/s41467-023-39287-7)
Supplement: Supplementary file 12 — Reporting Summary [file 41467_2023_39287_MOESM12_ESM.pdf]

nature portfolio

Corresponding author(s): Lars A. Akslen

Last updated by author(s): May 30, 2023

Reporting Summary

Nature Portfolio wishes to improve the reproducibility of the work that we publish. This form provides structure for consistency and transparency in reporting. For further information on Nature Portfolio policies, see our Editorial Policies and the Editorial Policy Checklist.

Statistics

For all statistical analyses, confirm that the following items are present in the figure legend, table legend, main text, or Methods section.

n/a	Confirmed	
☐	☒	The exact sample size (n) for each experimental group/condition, given as a discrete number and unit of measurement
☐	☒	A statement on whether measurements were taken from distinct samples or whether the same sample was measured repeatedly
☐	☒	The statistical test(s) used AND whether they are one- or two-sided<br>*Only common tests should be described solely by name; describe more complex techniques in the Methods section.*
☐	☒	A description of all covariates tested
☐	☒	A description of any assumptions or corrections, such as tests of normality and adjustment for multiple comparisons
☐	☒	A full description of the statistical parameters including central tendency (e.g. means) or other basic estimates (e.g. regression coefficient) AND variation (e.g. standard deviation) or associated estimates of uncertainty (e.g. confidence intervals)
☐	☒	For null hypothesis testing, the test statistic (e.g. F, t, r) with confidence intervals, effect sizes, degrees of freedom and P value noted<br>*Give P values as exact values whenever suitable.*
☒	☐	For Bayesian analysis, information on the choice of priors and Markov chain Monte Carlo settings
☒	☐	For hierarchical and complex designs, identification of the appropriate level for tests and full reporting of outcomes
☐	☒	Estimates of effect sizes (e.g. Cohen's d, Pearson's r), indicating how they were calculated

Our web collection on statistics for biologists contains articles on many of the points above.

Software and code

Policy information about availability of computer code

Data collection	LTQ-Orbitrap Elite, Tune 2.7.0 and Xcalibur 2.2<br>Q-Exactive HF, Tune 2.4 and Xcalibur 3.0<br>Exploris 480

Data analysis	MaxQuant v1.5.3.30 and v1.6.0.16<br>Perseus v1.6.0.2<br>Panther Classification System PANTHER v14.0, GO database released 2019-01-01<br>fgsea R-script v1.15.0<br>StringDB v10.5<br>Cytoscape v3.5.1<br>MCODE v1.4.2<br>Ingenuity Pathway Analysis (Qiagen)<br>SPSS v25<br>KM Plotter<br>J-Express v2012<br>Connectivity Map 02<br>Cibersort<br>SEEK<br>GSEA 4.3.2 (Broad Institute)<br>FragPipe v18 (with MSFragger v3.5, Philosopher v4.4 and Ionquant v1.8)<br>ComplexHeatmap v2.15.1 R-package

For manuscripts utilizing custom algorithms or software that are central to the research but not yet described in published literature, software must be made available to editors and reviewers. We strongly encourage code deposition in a community repository (e.g. GitHub). See the Nature Portfolio guidelines for submitting code & software for further information.

Data

Policy information about availability of data

All manuscripts must include a data availability statement. This statement should provide the following information, where applicable:

- Accession codes, unique identifiers, or web links for publicly available datasets
- A description of any restrictions on data availability
- For clinical datasets or third party data, please ensure that the statement adheres to our policy

The mass spectrometry proteomics data generated in this study have been deposited to the ProteomeXchange Consortium via the PRIDE partner repository (http://www.ebi.ac.uk/pride). The secretome data for the discovery panel of BCCLs are available via ProteomeXchange with the dataset identifier PXD027136. The microdissected patient material data are available via ProteomeXchange with identifier PXD027012. The secretome data for validation panel of BCCLs are available via ProteomeXchange with identifier PXD040532. Mass spectrometry data were searched against the forward and reverse Human UniProt database (https://www.uniprot.org/proteomes/UP000005640; downloaded/accessed 2016-01-08 (discovery BCCL panel), 2022-11-21 (validation BCCL panel), 2017-10-22 (microdissected patient material)). Clinical data on patients used for tissue microdissection might be made available for researchers on a request that does not include revelation of identifiable patient information, upon completion of a Data Transfer Agreement and confirmation of ethical approval. This study included analysis of data from the publicly available METABRIC-Discovery cohort (available from the European Genome-Phenome Archive, Dataset ID: EGAD00010000210; unique identifier: https://doi.org/10.1038/nature10983), and the proteomic dataset from Asleh et al. Nature Communications (2022) available from the supplementary information (unique identifier: https://doi.org/10.1038/s41467-022-28524-0). Survival analysis for hypoxia signatures was performed using the online KMplotter analysis platform – unique identifiers: (https://kmplot.com/analysis/). Publicly available data from the Cancer Cell Line Encyclopedia (CCLE) was used in this study. Processed transcriptomic data from breast cancer cell lines are available from CCLE and they are accessible via the depmap portal (https://depmap.org/portal/download/all/). CCLE proteomic data are available from https://gygi.hms.harvard.edu/publications/ccle.html. CIBERSORT analysis data from Craven et al. are available from https://github.com/kelgalla/tnbctils or https://doi.org/10.5281/zenodo.4542590. The SEEK database (https://seek.princeton.edu/seek/) was used for search-based exploration of the identified proteins (publicly available datasets: GSE45255.GPL96, unique identifiers: https://doi.org/10.1186/gb-2013-14-4-r34; GSE4922.GPL96, unique identifiers: https://doi.org/10.1158/0008-5472.CAN-05-4414; GSE22093.GPL96, unique identifiers: https://doi.org/10.1093/jnci/djq524, https://doi.org/10.1371/journal.pone.0049529; GSE15852.GPL96, unique identifiers: https://doi.org/10.1016/j.prp.2009.11.006). The remaining data are available within the article, supplementary information and source data file.

Research involving human participants, their data, or biological material

Policy information about studies with human participants or human data. See also policy information about sex, gender (identity/presentation), and sexual orientation and race, ethnicity and racism.

Reporting on sex and gender	Tumor tissues were collected from female patients. Sex was defined by the national and unique 11-digit personal identification number.
Reporting on race, ethnicity, or other socially relevant groupings	Not applicable.
Population characteristics	Tumor tissues were collected from patients (aged 50–69 years) diagnosed with breast carcinoma NST (no special type) during 1996-2003, as part of a prospective and population-based screening program.
Recruitment	The study did not include recruitment.
Ethics oversight	For the in-house human tumor samples used in our study (for microdissection and proteomics, n=24; for immunohistochemistry, n=42), the protocol was approved by the Western Regional Committee for Medical and Health Research Ethics, REC West (REK #2014/1984). The informed consent was waived by the REC West Committee, based on national guidelines, as well as the age and size of the full cohort covered by the approval. However, the actual patients included were informed about the research project and the possibility to withdraw. All studies were performed in accordance

with guidelines and regulations by the University of Bergen and REK, and in accordance with the Declaration of Helsinki Principles.

Note that full information on the approval of the study protocol must also be provided in the manuscript.

Field-specific reporting

Please select the one below that is the best fit for your research. If you are not sure, read the appropriate sections before making your selection.

☒ Life sciences ☐ Behavioural & social sciences ☐ Ecological, evolutionary & environmental sciences

For a reference copy of the document with all sections, see nature.com/documents/nr-reporting-summary-flat.pdf

Life sciences study design

All studies must disclose on these points even when the disclosure is negative.

Sample size	Sample size of the microdissected patient material (tumor stroma, n=24; tumor epithelium, n=24) were determined as a compromise between time consumption and biological samples needed to capture differences between groups (basal-like and luminal-like). Sample size of breast cancer cell lines (n=12) was also based on a compromise between maximum data collection and feasibility in generation of biological material from distinct conditions (normoxic and hypoxic conditions) as well as differences between groups (basal-like and luminal-like).
Data exclusions	In line with pre-determined criteria, proteins with missing values in more than 50% of samples in all groups (basal-like; luminal-like) were excluded to ensure data quality of discovery hypoxia secretome dataset. In the validation hypoxia secretome dataset, we did not apply these criteria to filter proteins.
Replication	Cell line work was done in triplicates for discovery hypoxia secretome experiments, and in duplicates for validation hypoxia secretome experiments. The expanded validation panel of breast cancer cell lines validated findings from discovery panel of breast cancer cell lines.
Randomization	Digested protein samples were analyzed by mass spectrometry in random order to avoid bias caused by instrument drift. In the validation experiment, hypoxia and normoxia samples were analyzed alternately.
Blinding	Operator of laser microdissector were blinded to the breast cancer subtypes (basal-like; luminal-like). Immunohistochemical staining was scored by researchers blinded to breast cancer molecular subtype and 33P signature score.

Reporting for specific materials, systems and methods

We require information from authors about some types of materials, experimental systems and methods used in many studies. Here, indicate whether each material, system or method listed is relevant to your study. If you are not sure if a list item applies to your research, read the appropriate section before selecting a response.

Materials & experimental systems

n/a	Involved in the study	
☐	☒	Antibodies
☐	☒	Eukaryotic cell lines
☒	☐	Palaeontology and archaeology
☒	☐	Animals and other organisms
☐	☒	Clinical data
☒	☐	Dual use research of concern
☒	☐	Plants

Methods

n/a	Involved in the study	
☒	☐	ChIP-seq
☒	☐	Flow cytometry
☒	☐	MRI-based neuroimaging

Antibodies

Antibodies used	Monoclonal rabbit antibody against NRF2 (ab62352, clone EP1808Y, Abcam), diluted 1:100
Validation	The NRF2 antibody was validated by the manufacturers in both positive and negative expressing cells (HELA) and tissue samples (human pancreatic carcinoma and human kidney cancer tissue) with known localization patterns to confirm specificity and sensitivity, and in-house breast cancer and placenta tissues were established as positive controls.

Eukaryotic cell lines

Policy information about cell lines and Sex and Gender in Research

Cell line source(s)	BT-474 (ATCC HTB-20)<br>MCF7 (ATCC HTB-22)<br>Hs 578T (ATCC HTB-126)

	MDA-MB-231 (ATCC HTB-26)<br>HCC1428 (ATCC CRL-2327)<br>T-47D (ATCC HTB-133)<br>ZR-75-1 (ATCC CRL-1500)<br>ZR-75-30 (ATCC CRL-1504)<br>MDA-MB-468 (ATCC HTB-132)<br>BT-549 (ATCC HTB-122)<br>HCC1143 (ATCC CRL-2321)<br>HCC1187 (ATCC CRL-2322)
Authentication	Not authenticated in-house, certificate of analysis from source.
Mycoplasma contamination	All cell lines tested negative for mycoplasma contamination.
Commonly misidentified lines<br>(See ICLAC register)	No commonly misidentified cell lines were used.

Clinical data

Policy information about clinical studies

All manuscripts should comply with the ICMJE guidelines for publication of clinical research and a completed CONSORT checklist must be included with all submissions.

Clinical trial registration	N/A
Study protocol	For the in-house human tumor samples used in our study (for microdissection and proteomics, n=24; for immunohistochemistry, n=42), the protocol was approved by the Western Regional Committee for Medical and Health Research Ethics, REC West (REK #2014/1984). The informed consent was waived by the REC West Committee, based on national guidelines, as well as the age and size of the full cohort covered by the approval. However, the actual patients included were informed about the research project and the possibility to withdraw. All studies were performed in accordance with guidelines and regulations by the University of Bergen and REK, and in accordance with the Declaration of Helsinki Principles.
Data collection	Tumor tissues were collected from female patients (aged 50–69 years) diagnosed with breast carcinoma NST (no special type) during 1996-2003, as part of a prospective and population-based screening program. Data was collected by mass spectrometry analysis.
Outcomes	No outcome data was used.
